# Associations of accelerometer-measured physical activity and physical activity-related cancer incidence in older women: results from the WHI OPACH Study

**DOI:** 10.1038/s41416-020-0753-6

**Published:** 2020-03-05

**Authors:** Humberto Parada, Emily McDonald, John Bellettiere, Kelly R. Evenson, Michael J. LaMonte, Andrea Z. LaCroix

**Affiliations:** 1 Division of Epidemiology and Biostatistics, School of Public Health, San Diego State University, San Diego, CA USA; 2 Moores Cancer Center, University of California, San Diego, La Jolla, CA USA; 3 Department of Family Medicine and Public Health, University of California, San Diego, San Diego, CA USA; 4 Department of Epidemiology, Gillings School of Global Public Health, University of North Carolina at Chapel Hill, Chapel Hill, NC USA; 5 Department of Epidemiology and Environmental Health, University at Buffalo, Buffalo, NY USA

**Keywords:** Cancer epidemiology, Cancer prevention, Risk factors

## Abstract

**Background:**

We examined the associations between accelerometry-measured physical activity (PA) and incidence of 13 cancers among a cohort of postmenopausal women.

**Methods:**

In this prospective study, 6382 women wore ActiGraph GT3X+ accelerometers at the hip for up to 7 days during 2012–2013, and were followed over a median of 4.7 years for diagnosis of 13 invasive cancers. Calibrated intensity cut points were used to define minutes per day of total, light and moderate-to-vigorous PA. We used multivariable Cox regression to estimate hazard ratios (HRs) and 95% confidence intervals (CIs) for tertiles, and one-standard deviation (SD) unit increments of PA exposures in relation to cancer incidence. We examined effect measure modification by age, race/ethnicity, body mass index and smoking history.

**Results:**

The highest (vs. lowest) tertiles of total, light and moderate-to-vigorous PA were associated with covariate-adjusted HRs of 0.72 (95% CI = 0.53–0.97), 0.81 (95% CI = 0.60–1.09) and 0.66 (95% CI = 0.48–0.91), respectively. In age-stratified analyses, HRs for total PA were lower among women <80 years (HR_per one-SD_ = 0.75, 95% CI = 0.63–0.90) than among women ≥80 years (HR_per one-SD_ = 0.99, 95% CI = 0.82–1.18) (*P*_Interaction_ = 0.03). Race/ethnicity, BMI and smoking did not strongly modify these associations.

**Conclusions:**

Engaging in physical activity may play a beneficial role in the prevention of certain cancers in older women.

## Background

Cancer is the second leading cause of death among women in the United States (US).^1^ In 2019, ~890,000 women will be diagnosed with invasive cancer, including 268,000 with breast cancer.^2^ Furthermore, shifting population demographics has led to a greater number of adults aged 65 and older,^3^ and thus an increased number of cancer patients and survivors, and growing cancer care expenditures.^4^ Given the high burden of cancer in the US, prevention is a significant public health issue.

More than half of the newly diagnosed cancer cases are considered preventable through lifestyle modifications, vaccinations and better implementation of clinical screening guidelines.^5^ Lifestyle factors, such as physical inactivity, are important contributors to the incidence of cancer.^6^ Although the exact biological mechanisms continue to be elucidated, it has long been hypothesised that physical activity (PA) may play an important role in cancer prevention by favourably impacting metabolic and hormonal pathways, and optimising physiologic and immunologic function in the human body, among other benefits.^7^ Numerous epidemiologic studies have shown that individuals who self-report higher levels of leisure-time PA have an overall lower risk of developing^8^ and dying from cancer.^9^ However, the benefits of PA for primary prevention appear to vary by cancer type. In a pooled analysis of 1.44 million adults from 12 prospective US and European cohorts, Moore et al. examined the relationship between leisure-time PA and incidence of 26 site-specific cancers, and reported that regularly engaging in higher levels of moderate-to-vigorous physical activity (MVPA) was associated with lower risk of 13 cancers; relative risks reported ranged from 0.58 for oesophageal adenocarcinoma to 0.90 for breast cancer.^10^ As noted by Moore et al., prospective cohort studies of PA and cancer incidence have largely relied on self-reported measures of PA.^10^ While self-reported assessment of PA is relatively inexpensive and easily employed in large epidemiologic studies, self-reported PA may result in measurement error, thus potentially biasing risk estimates, underscoring the need for using objective methods of PA measurement when feasible.^11,12^

In this study, we examined the association between PA assessed by accelerometry and incidence of 13 cancers previously shown to be associated with PA,^10^ among postmenopausal women participating in the Women’s Health Initiative (WHI) Objective Physical Activity and Cardiovascular Health (OPACH) Study.^13^ Furthermore, we examined effect measure modification by age, race/ethnicity, body mass index and smoking history, given the lack of available evidence on the association between accelerometry-measured PA and cancer incidence by these subgroups.^14^

## Methods

### Study population

The OPACH study, an ancillary to the Women’s Health Initiative (WHI), is a prospective investigation of accelerometer-measured PA and chronic disease outcomes among 7048 postmenopausal women aged 63–99 years who were originally enrolled in the WHI Long Life Study. Details of OPACH participant recruitment, enrolment and study procedures have been reported previously.^13^ In this study, from the 7048 women enrolled, we excluded 327 women who did not return an accelerometer, 232 women who returned accelerometers with unusable data and 107 women who did not meet our criteria for adherence, which was having at least 1 day with at least 10 h of awake accelerometer wear time, the conventional standard for compliant wear, as described below. These exclusions resulted in an analytic sample of 6382 women. Of these, the majority (97.6%) had ≥3 adherent days (mean = 6.3, median = 7.0 days). At OPACH baseline, women had a mean age of 78.7 years (SD = 6.7 years) and a mean BMI of 28.2 kg/m^2^. The majority of women were well-educated as 79.7% reported attending college or receiving a college degree, and 49.4% self-reported their race/ethnicity as non-Hispanic white, as previously reported.^13,15^ All study protocols were approved by Fred Hutchinson Cancer Research Center Institutional Review Board, and all women gave written informed consent.

### Cancer incidence

Cancer incidence was defined as a composite outcome variable that included 13 site-specific fatal and non-fatal invasive cancers previously shown to be inversely associated with MVPA in a general population of adults including oesophageal, liver, lung, kidney, gastric, endometrial, leukaemia, multiple myeloma, colon, head and neck, rectal, bladder and breast cancers.^10^ Physician-adjudicated cancer diagnosis was ascertained from enrolment in 2012–2013 through March 31, 2018, by ongoing surveillance in the national WHI program using annual mailed health updates, reports by family or friends, review of medical records, obituaries and linkage to the National Death Index. We censored follow-up time on the date of PA-related cancer diagnosis, the date of death from an event unrelated to cancer or the date of last contact. Excluding women with a cancer diagnosis prior to OPACH baseline would prevent the assessment of how PA is associated with the first-time occurrence of previously undiagnosed cancers, an important public health question given the large number of older adults with prevalent cancer. To assess whether having a previous cancer diagnosis impacted the results, we performed sensitivity analyses that excluded women with a history of cancer diagnosis at OPACH baseline (*n* = 1071). History of cancer diagnosis at WHI enrolment (1993–1998) was assessed by self-report, and at OPACH baseline, history of cancer between WHI enrolment and OPACH baseline was assessed by self-reports that were adjudicated by physicians using medical record review.

### Physical activity

PA was measured using the ActiGraph GT3X+ triaxial accelerometer. Participants were instructed to wear the accelerometer at their right hip during all waking hours, except when bathing or swimming, and while sleeping, for 7 days. Data measured at 30 Hz were aggregated to 15-s epochs using the normal filter in ActiLife software (v6), and the three axes were converted into a measure of the vector magnitude that was used to operationalise activity intensity.^16^ A commonly used computer-based automated algorithm was used to determine accelerometer wear time with a window of 90 min, a streamframe of 30 min and a tolerance of 2 min.^17^ Times that the participants were out of bed were determined by using data from sleep logs completed for each night of accelerometer wear.^17^ Missing sleep log data were imputed using person-specific means, if available, or the sample mean. Previously calibrated^16^ cut points were then used to assess total PA (defined as movement resulting in energy expenditure ≥1.6 METs, the cut point for sedentary behaviour), light PA (movement resulting in energy expenditure between 1.6 and 2.9 METs) and MVPA (movement resulting in energy expenditure ≥3.0 METs). Total PA was then measured as the average minutes per day with accelerometer counts per 15 s of ≥19. Light PA and MVPA were measured as the average minutes per day with accelerometer counts per 15 s of 19–518 and ≥519, respectively.

### Covariates

Information regarding baseline covariates was collected using self-report questionnaires and anthropometric measurements from an in-home clinical examination. Covariates included awake accelerometer wear time (hours per day), age (continuous in years), race/ethnicity (non-Hispanic white, non-Hispanic black and Hispanic), education level (high school or less, some college and college graduate), a composite measure of smoking status and pack-years (never smoker, former smoker/≤7.5 or >7.5 pack-years and current smoker/≤22.5 or >22.5 pack-years), frequency of alcohol intake (non-drinker, <1, 1–4 and 5–7 drinks per week), measured height and weight to calculate body mass index (BMI, in kg/m^2^), hormone therapy use (no, yes), self-rated health status (excellent or very good, good, fair or poor), number of chronic conditions including history of coronary heart disease, stroke, diabetes mellitus, osteoarthritis, depression, chronic obstructive pulmonary disease, cognitive impairment, frequent falls, hearing loss or visual impairment (none, 1–2 and 3+), diet quality assessed using the Health Eating Index (HEI) 2010 (quartiles, with higher quartiles indicating greater intake of foods that align with key dietary recommendations)^18,19^ and history of cancer diagnosis (no, yes).

### Statistical analysis

We standardised the minutes of total, light and moderate-to-vigorous PA for differences in wear time using the residuals obtained when regressing each PA variable on wear time, and then created tertiles of wear time-standardised PA variables for use in statistical analyses.^20–22^ Cut points used to define tertiles were ≤292.20, 292.21–371.34 and >371.34 min per day for total PA; ≤251.76, 251.77–315.02 and >315.02 min per day for light PA; ≤30.83, 30.84–57.52 and >57.52 min per day for MVPA.

We used Kaplan–Meier survival curves to inspect the unadjusted associations between tertiles of total, light and moderate-to-vigorous PA with the composite incident PA-related cancer outcome. We examined Schoenfeld residuals to assess the proportional hazards assumption. There were no appreciable violations of the proportional hazards assumption when we compared the survival curves for the highest and lowest tertiles of PA. We used Cox regression to estimate hazard ratios (HRs) and 95% confidence intervals (CIs) for tertiles of total, light and moderate-to-vigorous PA in association with incidence of invasive cancer. We also examined linear trends (i.e., *P*_Trend_) using continuous minutes of wear time-standardised PA. Cox regression models were first adjusted for age (Model 1). We then included additional adjustment for race/ethnicity, education, smoking status, hormone replacement use, self-assessed health status and number of comorbidities (Model 2). Last, we included adjustment for BMI (Model 3), a presumed mediator of the association between PA and cancer incidence.

We used imputation to account for missing covariate data. Variables with high proportions of missing values included HEI-2010 (21.9% missing), alcohol use (16.3% missing) and smoking status/pack-years (12.3% missing). We imputed missing values using SPSS, which employs a fully conditional specification algorithm, an iterative Markov Chain Monte Carlo procedure that sequentially imputes missing values.^23^ SPSS applies logistic or multinomial logistic regression to categorical variables, and linear regression to continuous-scale variables. We used 25 imputations with 500 iterations, and included sociodemographic (age and race/ethnicity, education and income), lifestyle and behavioural factors (smoking status, smoking pack-years, alcohol use, body mass index, hormone therapy use and HEI-2010), health characteristics (self-rated health, number of comorbid conditions and history of cancer), wear time-standardised minutes of total, light and moderate-to-vigorous PA, the cancer event indicator and the Nelson–Aalen estimator of the cumulative hazard.^24^ In sensitivity analyses, we also excluded women who were diagnosed or censored within 6 months of follow-up, and examined the associations between PA and overall cancer incidence. We also examined whether our composite measure of cancer was robust to the range of cancers included by removing each of the 13 site-specific cancers at a time in fully adjusted models examining the PA measures continuously. Last, we used multivariable Cox regression isotemporal substitution models to estimate the effect of statistically replacing time from one activity for an equal amount of time from another activity. As reported in the isotemporal literature,^25–28^ we first divided continuous minutes of sedentary time, light PA and MVPA by 30, so that a unit increase in sedentary behaviour or activity represented an increase of 30 min per day within the given category. We then entered all variables that characterise total activity wear time and total wear time into a single model, and sequentially dropped each one of the activity variables.

In addition to examining PA tertiles, we analysed PA continuously per standard deviation unit increments according to the overall cohort, and a priori strata of interest defined by age (<80 vs. ≥80 years), race/ethnicity (white, black and Hispanic), BMI (<30 vs. ≥30 kg/m^2^) and smoking history (ever vs. never smoker). Tests for multiplicative interaction were evaluated using likelihood ratio tests comparing the fully adjusted Cox regression models with cross-product terms for continuous PA and each of the categorical covariates, with the reduced model without the interaction terms.

All statistical analyses were performed using IBM SPSS Statistics Version 26 (IBM Corp., Armonk, NY).

## Results

Over a median follow-up of 4.7 years (max = 6.0 years), among the 6382 women in the cohort there were 1188 incident cancers, of which 272 were PA-related. This included 99 invasive breast cancers, 52 lung cancers, 33 colorectal cancers, 20 leukaemias, 17 bladder cancers, 16 myelomas, 10 endometrial cancers, 7 kidney cancers, 6 liver cancers, 5 stomach cancers, 5 head and neck cancers and 2 oesophageal cancers. Approximately 2.7% of women (*n* = 171) in this cohort were lost to follow-up, and were censored for lack of outcome data with partial follow-up. At baseline, the women engaged in a mean 334.1 (SD = 90.8) min per day of total PA, including a mean 284.4 (SD = 72.8) min per day of light PA, and 49.7 (SD = 33.3) min per day of MVPA. Overall, women with higher versus lower levels of wear time-standardised total PA were younger, and had lower BMI and higher levels of alcohol intake, self-rated general health and diet quality, and had no history of cancer diagnosis (Table 1).

**Table 1:** OPACH participant baseline characteristics by tertiles of wear time-standardised total physical activity.

Characteristic	Total PA minutes per day mean (SD)	Tertiles of total PA		
		T1 (low) *n* (%)	T2 *n* (%)	T3 (high) *n* (%)
*N*	6382	2,125	2,126	2,131
Age (years)				
60–69	369.8 (88.2)	127 (6.0)	213 (10.0)	314 (14.7)
70–79	348.9 (90.5)	692 (32.6)	856 (40.3)	1009 (47.3)
80–89	317.1 (87.4)	1162 (54.7)	972 (45.7)	772 (36.2)
≥90	288.2 (79.5)	144 (6.8)	85 (4.0)	36 (1.7)
Race/ethnicity				
Non-Hispanic white	318.4 (89.5)	1266 (59.6)	1039 (48.9)	845 (39.7)
Non-Hispanic black	341.9 (88.9)	636 (29.9)	736 (34.6)	779 (36.6)
Hispanic/Latina	364.0 (89.2)	223 (10.5)	351 (16.5)	507 (23.8)
Highest education level				
High school or less	337.0 (90.3)	409 (19.4)	420 (20.0)	460 (21.6)
Some college	329.7 (91.4)	875 (41.5)	810 (38.5)	771 (36.3)
College graduate or more	337.2 (90.7)	826 (39.1)	873 (41.5)	895 (42.1)
Missing		15	23	5
Smoking status and pack-years				
Never	336.7 (90.17)	996 (54.2)	1077 (57.6)	1067 (56.5)
Former				
≤7.5 pack-years	342.0 (90.43)	337 (18.3)	393 (21.0)	426 (22.6)
>7.5 pack-years	325.7 (91.74)	435 (23.7)	350 (18.7)	356 (18.9)
Current				
≤22.5 pack-years	324.0 (85.42)	30 (1.6)	34 (1.8)	20 (1.1)
>22.5 pack-years	291.7 (83.98)	41 (2.2)	17 (0.9)	19 (1.0)
Missing		286	255	243
Alcohol intake in the past 3 months				
Non-drinker	329.9 (89.5)	681 (41.0)	666 (36.8)	618 (33.1)
<1 drink per week	334.1 (89.8)	598 (36.0)	646 (35.7)	601 (32.2)
1–4 drinks per week	353.3 (87.3)	235 (14.1)	310 (17.1)	392 (21.0)
5–7 drinks per week	355.4 (92.3)	149 (9.0)	187 (10.3)	257 (13.8)
Missing		462	317	263
Body mass index (kg/m^2^)				
<18.5	381.5 (96.5)	14 (0.7)	24 (1.2)	43 (2.1)
18.5–24.9	363.2 (91.3)	414 (21.1)	581 (29.2)	850 (42.1)
25.0–29.9	335.2 (84.3)	681 (34.8)	773 (38.8)	702 (34.7)
30.0–34.9	312.4 (85.2)	473 (24.1)	392 (19.7)	290 (14.3)
35.0–39.9	301.9 (87.2)	240 (12.3)	146 (7.3)	105 (5.2)
≥40.0	283.3 (82.4)	137 (7.0)	76 (3.8)	31 (1.5)
Missing		166	134	110
Self-rated general health				
Excellent or very good	347.2 (90.7)	888 (42.0)	1071 (50.6)	1237 (58.3)
Good	325.2 (87.6)	938 (44.3)	852 (40.2)	740 (34.9)
Fair or poor	303.1 (92.1)	289 (13.7)	195 (9.2)	146 (6.9)
Missing		10	8	8
Number of chronic conditions				
0	355.2 (92.1)	275 (13.0)	356 (16.8)	474 (22.3)
1	342.9 (89.7)	635 (30.0)	734 (34.7)	781 (36.8)
2	330.0 (87.8)	594 (28.1)	578 (27.4)	534 (25.2)
≥3	308.8 (89.0)	611 (28.9)	445 (21.1)	334 (15.7)
Missing		10	13	8
Any hormone replacement therapy				
No	333.2 (90.8)	2094 (98.5)	2072 (97.5)	2054 (96.4)
Yes	367.9 (88.3)	31 (1.5)	54 (2.5)	77 (3.6)
Healthy Eating Index 2010				
Quartile 1	323.4 (91.2)	472 (29.5)	400 (23.7)	374 (22.0)
Quartile 2	334.0 (88.8)	406 (25.4)	445 (26.3)	397 (23.4)
Quartile 3	337.8 (89.1)	391 (24.5)	421 (24.9)	435 (25.6)
Quartile 4	350.3 (88.6)	329 (20.6)	425 (25.1)	492 (29.0)
Missing		527	435	433
History of cancer diagnosis				
No	337.15 (90.8)	1706 (80.3)	1762 (82.9)	1843 (86.5)
Yes	318.71 (89.7)	419 (19.7)	364 (17.1)	288 (13.5)

WHI OPACH participants enrolled in 2012–2013 and followed up for cancer incidence through March 31, 2018.

As shown in Table 2, incident PA cancer HRs declined in a dose–response pattern across increasing tertiles of total PA. Women with the highest (vs. lowest) tertiles of total PA had a HR of 0.72 (95% CI = 0.53–0.97) in Model 2 (*P*_Trend_ = 0.03). Additional adjustment for BMI slightly attenuated this association (Model 3 HR = 0.76, 95% CI = 0.55–1.04, *P*_Trend_ = 0.10). The inverse association between PA and cancer incidence was more pronounced when we considered MVPA. The highest (vs. lowest) tertile of MVPA was associated with a HR of 0.66 (95% CI = 0.48–0.91) in Model 2 (*P*_Trend_ = 0.03), with some attenuation in the HR after BMI adjustment (Model 3 HR = 0.69, 95% CI = 0.50–0.95, *P*_Trend_ = 0.05). Patterns of association for light PA were similar to those of MVPA; however, estimates for light PA were closer to the null.

**Table 2:** Hazard ratios (HRs) and 95% confidence intervals (CIs) for physical activity-related cancer incidence and tertiles of wear time-standardised total, light and moderate-to-vigorous physical activity (*N* = 6382).

Physical activity	Tertiles of PA			
**Total physical activity**	**Tertiles of total PA**			***P***_**Trend**_^a^
	**T1 (low)**	**T2**	**T3 (high)**	
Total PA minutes per day, mean (SD)	236.0 (43.5)	331.7 (22.8)	434.1 (52.0)	
Cancer events, *n* (%)	110 (5.2)	84 (4.0)	78 (3.7)	
Person-years	9,014.17	9,518.96	9,741.07	
Incidence rate per 1,000 person-years	12.20	8.82	8.01	
*Model 1***:** Hazard ratio (95% CI)^b^	1.00	0.73 (0.55–0.98)	0.67 (0.50–0.90)	0.01
*Model 2***:** Hazard ratio (95% CI)^c^	1.00	0.78 (0.58–1.04)	0.72 (0.53–0.97)	0.03
*Model 3***:** Hazard ratio (95% CI)^d^	1.00	0.80 (0.60–1.07)	0.76 (0.55–1.04)	0.10
**Light physical activity**	**Tertiles of light PA**			***P***_**Trend**_^a^
	**T1 (low)**	**T2**	**T3 (high)**	
Light PA minutes per day, mean (SD)	205.5 (35.8)	283.3 (18.1)	364.2 (40.9)	
Cancer events, *n* (%)	104 (4.9)	86 (4.0)	82 (3.8)	
Person-years	9092.91	9522.33	9659.0	
Incidence rate per 1000 person-years	11.44	9.03	8.49	
*Model 1***:** Hazard ratio (95% CI)^b^	1.00	0.80 (0.60–1.07)	0.76 (0.57–1.02)	0.03
*Model 2***:** Hazard ratio (95% CI)^c^	1.00	0.85 (0.63–1.13)	0.81 (0.60–1.09)	0.10
*Model 3***:** Hazard ratio (95% CI)^d^	1.00	0.88 (0.66–1.19)	0.86 (0.63–1.18)	0.25
**Moderate-to-vigorous physical activity**	**Tertiles of MVPA**			***P***_**Trend**_^a^
	**T1 (low)**	**T2**	**T3 (high)**	
MVPA minutes per day, mean (SD)	18.3 (8.7)	43.3 (7.4)	87.4 (27.3)	
Cancer events, *n* (%)	113 (5.3)	79 (3.7)	80 (3.8)	
Person-years	8953.23	9501.14	9819.8	
Incidence rate per 1000 person-years	12.62	8.31	8.15	
*Model 1***:** Hazard ratio (95% CI)^b^	1.00	0.66 (0.49–0.89)	0.65 (0.48–0.88)	0.02
*Model 2***:** Hazard ratio (95% CI)^c^	1.00	0.67 (0.50–0.90)	0.66 (0.48–0.91)	0.03
*Model 3***:** Hazard ratio (95% CI)^d^	1.00	0.68 (0.50–0.91)	0.69 (0.50–0.95)	0.05

WHI OPACH participants enrolled in 2012–2013 and followed up for cancer incidence through March 31, 2018. Women were censored at the end of follow-up, at the last contact or at the time of death from an event unrelated to cancer.

*BMI* body mass index, *MVPA* moderate-to-vigorous physical activity, *PA* physical activity, *SD* standard deviation.

^a^*P*-values from chi-square tests for linear trend using Cox regression models with continuous PA variables.

^b^Model 1 is adjusted for age (years).

^c^Model 2 is adjusted for age (years), race/ethnicity (non-Hispanic white, non-Hispanic black and Hispanic), education (≤high school, some college and ≥college), smoking status/pack-years (never smoker, former smoker/≤7.5 or >7.5 pack-years and current smoker/≤22.5 or >22.5 pack-years), alcohol intake (non-drinker, <1 drink per week, 1–4 drinks per week and 5–7 drinks per week), hormone replacement therapy (no, yes), self-rated general health (excellent or very good, good, fair or poor), number of comorbid conditions (0, 1, 2, 3+), HEI-2010 (quartiles) and history of cancer diagnosis (no, yes).

^d^Model 3 is adjusted for age (years), race/ethnicity (non-Hispanic white, non-Hispanic black and Hispanic), education (≤high school, some college and ≥college), smoking status/pack-years (never smoker, former smoker/≤7.5 or >7.5 pack-years and current smoker/≤22.5 or >22.5 pack-years), alcohol intake (non-drinker, <1 drink per week, 1–4 drinks per week and 5–7 drinks per week), hormone replacement therapy (no, yes), self-rated general health (excellent or very good, good, fair or poor), number of comorbid conditions (0, 1, 2, 3+), HEI-2010 (quartiles), history of cancer diagnosis (no, yes) and BMI (<18.5, 18.5–24.9, 25.0–29.9, 30–34.9, 35–39.9 and ≥40 kg/m^2^).

In Table 3, we present the results for PA-related cancer incidence and continuous PA per one-standard deviation (SD) unit increments of total, light and moderate-to-vigorous PA for the overall cohort, and stratified by age, race/ethnicity, BMI and smoking history. Among women <80 years, a one-SD unit increase in total PA was associated with a multivariable HR of 0.75 (95% CI = 0.63–0.90); however, this association was null among women ≥80 years (HR = 0.99, 95% CI = 0.82–1.18) (*P*_Interaction_ = 0.03). This interaction was also evident for light PA in which the HR among women <80 years was 0.78 (95% CI = 0.65–0.93) and 1.01 (95% CI = 0.85–1.20) among women ≥80 years (*P*_Interaction_ = 0.04), but less apparent for MVPA (*P*_Interaction_ = 0.31). There was a suggestion of effect measure modification by smoking history for MVPA and PA-related cancer incidence (*P*_Interaction_ = 0.09), with a stronger inverse association among never smokers (HR = 0.76, 95% CI = 0.61–0.96) than among ever smokers (HR = 0.89, 95% CI = 0.74–1.08) (Table 3). Race/ethnicity and BMI did not modify these associations.

**Table 3:** Hazard ratios (HR) and 95% confidence intervals (CI) for physical activity-related cancer incidence and standardised total, light and moderate-to-vigorous physical activity in the cohort overall and among cohort subgroups (*N* = 6382).

Cohort group	Cancer events *n* (%)	Total PA^a^		Light PA^a^		MVPA^a^	
		HR (95% CI)^b^	*P*_Interaction_^c^	HR (95% CI)^b^	*P*_Interaction_^c^	HR (95% CI)^b^	*P*_Interaction_^c^
Overall	272 (4.3)	0.87 (0.76–0.99)		0.90 (0.79–1.02)		0.85 (0.74–0.98)	
Age (years)			0.03		0.04		0.31
<80	132 (4.1)	0.75 (0.63–0.90)		0.78 (0.65–0.93)		0.79 (0.65–0.96)	
≥80	140 (4.4)	0.99 (0.82–1.18)		1.01 (0.85–1.20)		0.92 (0.75–1.14)	
Race/ethnicity			0.45		0.39		0.80
Non-Hispanic white	146 (4.6)	0.93 (0.77–1.11)		0.96 (0.81–1.14)		0.89 (0.73–1.08)	
Non-Hispanic black	89 (4.1)	0.75 (0.60–0.94)		0.78 (0.63–0.98)		0.76 (0.58–0.99)	
Hispanic/Latina	37 (3.4)	0.89 (0.62–1.27)		0.92 (0.64–1.30)		0.89 (0.63–1.26)	
BMI (kg/m^2^)			0.38		0.20		0.81
<30	174 (4.0)	0.94 (0.80–1.11)		0.98 (0.83–1.15)		0.88 (0.74–1.04)	
≥30	98 (4.8)	0.81 (0.64–1.02)		0.82 (0.65–1.02)		0.86 (0.66–1.14)	
Cigarette-smoking history			0.24		0.48		0.09
Never	125 (3.6)	0.80 (0.65–0.97)		0.85 (0.70–1.02)		0.76 (0.61–0.96)	
Ever	147 (5.1)	0.90 (0.75–1.07)		0.92 (0.78–1.09)		0.89 (0.74–1.08)	

WHI OPACH participants enrolled in 2012–2013 and followed up for cancer incidence through March 31, 2018. Women were censored at the end of follow-up, at the last contact or at the time of death from an event unrelated to cancer.

*BMI* body mass index, *MVPA* moderate-to-vigorous physical activity, *PA* physical activity.

^a^One-standard deviation unit increment of total PA = 90.8 min, light PA = 72.8 min and MVPA = 33.3 min.

^b^Model is adjusted for age (years), race/ethnicity (non-Hispanic white, non-Hispanic black and Hispanic), education (≤high school, some college and ≥college), smoking status/pack-years (never smoker, former smoker/≤7.5 or >7.5 pack-years and current smoker/≤22.5 or >22.5 pack-years), alcohol intake (non-drinker, <1 drink per week, 1–4 drinks per week and 5–7 drinks per week), hormone replacement therapy (no, yes), self-rated general health (excellent or very good, good, fair or poor), number of comorbid conditions (0, 1, 2, 3+), HEI-2010 (quartiles) and history of cancer diagnosis (no, yes), as appropriate.

^c^Interaction evaluated using likelihood ratio tests comparing Cox regression models with cross-product terms for continuous physical activity, and categorical covariates to reduced models without the interaction terms.

We conducted several sensitivity analyses to examine the robustness of our findings reported here. Excluding women with a cancer diagnosis prior to OPACH enrolment (Supplementary Table 1), and excluding those who were diagnosed or censored within 6 months of follow-up (Supplementary Table 2), resulted in no appreciable differences in the HR estimates. In analyses in which we considered overall cancer incidence as the outcome, age-only adjusted associations were generally greater in magnitude for all physical activity categories. The HRs were, however, closer to the null with additional covariate adjustment than those in which we used PA-related cancer incidence as the outcome (Supplementary Table 3). The results for total PA, light PA and MVPA were similar to the overall results when we excluded each of the cancer sites at a time from the composite measure; the largest changes in the estimates (6–8%) were observed when we excluded lung cancers, with some loss in precision (Supplementary Table 4). Last, the results of the isotemporal substitution models indicated that replacing 30 min per day of sedentary time with 30 min of light PA or MVPA was associated with HRs of PA-related cancer incidence of 0.97 (95% CI = 0.92–1.03) and 0.89 (95% CI = 0.78–1.02), respectively.

## Discussion

In this large cohort study of postmenopausal women, examining accelerometer-measured PA, higher levels of total PA and, in particular, MVPA, were associated with reduced risk of 13 site-specific cancers. The mean estimate of MVPA in this study (50 min per day), while higher than that reported in the Women’s Health Study (mean = 28 min per day),^29^ was similar to the estimate reported in the British Heart Study (mean = 39 min per day)^30^ and lower than that reported in a validation study of accelerometer-measured PA in older adults (mean = 68 min per day).^31^ Although less pronounced and not statistically significant, the highest versus lowest tertile of light PA was also inversely associated with incidence of the 13 cancers of interest. Furthermore, the inverse associations for total and light PA with cancer incidence appeared to be stronger in women less than 80 years old as compared with women over 80 years; however, the association between MVPA and cancer incidence was not different in younger compared with older women.

Over the past decade or so, epidemiologic studies have utilised sensor devices (e.g., accelerometers) to quantify total and intensity-specific PA more accurately and comprehensively than is possible by questionnaire assessments.^32^ Associations with cardiometabolic risk factors, all-cause and cardiovascular mortality outcomes have been published frequently. In a previous OPACH study, accelerometer-measured PA was not clearly associated with total cancer mortality (87 deaths over a mean 3.1-year follow-up).^15^ This study expands on the limited published findings on cancer outcomes and accelerometer-measured PA by showing clear and strong evidence of lower risk of 13 cancers associated with higher amounts of accelerometer-measured MVPA, even after controlling for several relevant risk predictors including BMI.

A large number of observational^8,9^ and clinical studies^33^ have reported the benefits of PA for reducing the risk of overall cancer incidence and cancer mortality. In a meta-analysis of 126 cohort studies, Liu et al. reported a relative risk of 0.90 for overall cancer incidence among individuals participating in the greatest amount of leisure-time PA (i.e., 95th percentile) as compared with those participating in the least amount of leisure-time PA (i.e., 5th percentile).^8^ In comparison, a meta-analysis by Li et al., which included 36 population-based studies from North America, Europe and Asia, reported a 16% decrease in cancer mortality, when compared with individuals who were physically inactive.^9^ Although these meta-analyses were able to pool data from millions of individuals, both relied on studies that used self-reported measures of PA, which have been shown to lack precision, often reflecting over- or under-reporting of activities that are challenging to assess by questionnaire,^11^ and fail to capture all activities performed throughout the day. Therefore, even these exceptionally large meta-analyses, when based on self-reported PA exposures, may not provide an accurate representation of the true association between PA and cancer incidence in the community.

It has long been hypothesised that engaging in PA, and avoiding sedentary behaviour, may play an important role in cancer prevention; however, the biological pathways regulating this observed risk reduction are still being clarified.^34,35^ Various site-specific cancers have different magnitudes of association with PA, suggesting that PA does not modulate one common pathway to carcinogenesis, but rather affects a number of biological processes, including metabolic profiles,^34–36^ hormone levels^34,35,37^ and immunologic functions,^34,38–40^ which in turn impact the risk of cancer over an individual’s lifespan.^33,35^

Our stratified results, if reproducible in other populations, suggest that PA may play a stronger role in cancer prevention in women up to at least the age of 80, while the beneficial impact of PA on cancer may wane at ages over 80. PA may be less effective in protecting against the likelihood of carcinogenesis, as the effects of oxidative stress and DNA damage continue to accumulate over the lifespan.^41,42^ Moreover, the biological pathways that protect the body from cancer tend to operate less efficiently with increasing age.^43^

The major strength of our study lies in the careful measurement of PA using triaxial accelerometers with output precisely calibrated to older women’s movement patterns and energy expenditure in a laboratory substudy.^16^ Thus, our study provides the first epidemiologic cohort study evidence that objectively measured PA is inversely associated with incidence of 13 cancers in community-dwelling older women in later life. Furthermore, the OPACH study has detailed information on relevant characteristics that could be related to both PA and cancer incidence, including demographics, lifestyle behaviours, personal medical history and anthropometric measures. The limitations of this study include the use of a composite cancer outcome comprising a relatively small number of incident cancer cases observed over the 4–6-year follow-up period, which precluded examination of site-specific cancers separately. In addition, because of the relatively short follow-up, the role that PA plays in cancer initiation within the 4–6 years prior to diagnosis is unclear. However, while the inferences we can make about cancer initiation may be limited, it is plausible that some cancers could develop within this timeframe. Furthermore, PA may also impact cancer risk by inhibiting tumour progression by, for example, suppressing tumour growth and angiogenesis, and stimulating apoptosis.^44^ Thus, PA likely exerts a combination of effects on both cancer initiation and progression, and the relative importance of each may depend on the site-specific cancer being considered and its inherent aggressiveness. The associations reported here, therefore, are still relevant for understanding how PA impacts cancer risk. Evaluation of a composite cancer incidence outcome also makes it challenging to ascribe biological mechanisms that might underpin the inverse associations observed herein, as the site-specific cancers identified during the present follow-up experience could have both shared as well as unique mechanisms through which PA could mitigate risk. Although our measure of PA was objective, we relied on a single assessment of PA, and so, were not able to account for changes in PA over time, which could be important in disentangling the influence that PA has on cancer occurrence from that which occult cancer potentially exerts on PA levels proximal to diagnosis, and while PA cut points were derived from a calibration study conducted for OPACH, the calibration study was laboratory-based only, and did not include free-living testing. We also acknowledge that although the accelerometer assessment provides an objective assessment of PA, it is not without limitations. Women may have altered their behaviour in response to wearing the device; however, this would have resulted in estimates that were biased towards the null, as it would have been non-differential with respect to cancer incidence. In addition, some activities such as swimming and bicycling are not well assessed by accelerometers. In the OPACH Study, 15% of women reported riding a bicycle or using a stationary bicycle, and 7% of women reported swimming regularly. This underestimate of PA is also likely to attenuate the results. In addition, there is high correlation between device wear time and each of the PA categories, and so it is difficult to separately estimate the associations of each of the PA categories. However, the results of our isotemporal substitution models suggest that statistically replacing sedentary time with MVPA had a stronger protective association with cancer incidence than replacing sedentary time with light PA. Another limitation of our study is that the study population is restricted to postmenopausal women, and thus it is unknown whether these findings can be generalised to younger women or men. Even so, the hazard ratio estimate of 0.66 for cancer incidence and MVPA is within the range of hazard ratios (0.58–0.91) reported by Moore et al. As with any observational study, there is the possibility that our findings are biased due to uncontrolled confounding, in particular, with regard to the covariate information relevant to the various site-specific cancers we included in the composite outcome variable, though, our results are consistent with the literature published to date. Last, although cancer diagnoses were physician-adjudicated, it is possible that we may have missed some cancers; however, this outcome misclassification is likely to be minimal, given the high commitment of the women participating in the WHI Study, as evidenced by the low proportion of women lost to follow-up, and the high degree of adherence to the accelerometer protocols.

## Conclusions

Our study supports the hypothesis that total PA, including time spent in moderate-to-vigorous-intensity activities, plays a beneficial role in the primary prevention of 13 site-specific cancers in older women. While the U.S. Department of Health and Human Services provides overarching PA guidelines for Americans, currently, there is insufficient evidence on the benefits of physical activity on cancer prevention among older adults^14^ whose PA needs may differ from the general population. Because of the known challenges to measuring PA using questionnaires, particularly in older adults and women, and because of the continued burden that cancer imparts on an aging society, if confirmed, our findings could have important implications to future guideline recommendations for cancer prevention in older adults.

## Supplementary information

Supplemental Material

## Data Availability

The datasets generated and/or analysed during this study are not publicly available, but may be available from WHI study on reasonable request.
