# Supplementary material for: Associations of accelerometer-measured physical activity and physical activity-related cancer incidence in older women: results from the WHI OPACH Study
Source: Br J Cancer. 2020 Mar 5;122(9):1409–16. doi: 10.1038/s41416-020-0753-6 (PMC7188876; doi:10.1038/s41416-020-0753-6)
Supplement: Supplementary file 1 — Supplemental Material [file 41416_2020_753_MOESM1_ESM.docx]

### **Supplemental Table 1**. Hazard ratios (HR) and 95% confidence intervals (CI) for physical activity-related cancer incidence and tertiles of wear time-standardized total, light, and moderate-to-vigorous physical activity, excluding women with a cancer diagnosis prior to OPACH baseline (*n*=5,311).

| **Total Physical Activity** | **Tertiles of Total PA** | | | ***P*_Trend_**^a^ |
| --- | --- | --- | --- | --- |
|  | **T1 (low)** | **T2** | **T3 (high)** |  |
| Total PA minutes per day, mean (SD) | 236.9 (42.5) | 332.1 (22.8) | 434.7 (52.3) |  |
|  |  |  |  |  |
| Cancer events, n (%) | 89 (5.2) | 60 (3.4) | 69 (3.7) |  |
| Person-years | 7,285.39 | 7,943.05 | 8,455.6 |  |
| Incidence rate per 1,000 person-years | 12.22 | 7.55 | 8.16 |  |
|  |  |  |  |  |
| Hazard ratio (95% CI)^b^ | 1.00 | 0.65 (0.47-0.91) | 0.71 (0.51-0.99) | 0.05 |
|  |  |  |  |  |
| **Light Physical Activity** | **Tertiles of Light PA** | | | ***P*_Trend_**^a^ |
|  | **T1 (low)** | **T2** | **T3 (high)** |  |
| Light PA minutes per day, mean (SD) | 206.2 (34.9) | 283.4 (18.1) | 365.2 (41.4) |  |
|  |  |  |  |  |
| Cancer events, n (%) | 85 (5.0) | 64 (3.6) | 69 (3.8) |  |
| Person-years | 7,385.49 | 8,003.09 | 8,295.43 |  |
| Incidence rate per 1,000 person-years | 11.51 | 8.00 | 8.32 |  |
|  |  |  |  |  |
| Hazard ratio (95% CI)^b^ | 1.00 | 0.73 (0.53-1.02) | 0.78 (0.56-1.08) | 0.09 |
|  |  |  |  |  |
| **Moderate-to-Vigorous Physical Activity** | **Tertiles of MVPA** | | | ***P*_Trend_**^a^ |
|  | **T1 (low)** | **T2** | **T3 (high)** |  |
| MVPA minutes per day, mean (SD) | 18.4 (8.6) | 43.3 (7.5) | 87.6 (27.3) |  |
|  |  |  |  |  |
| Cancer events, n (%) | 88 (5.1) | 61 (3.5) | 69 (3.7) |  |
| Person-years | 7,283.10 | 7,887.96 | 8,512.96 |  |
| Incidence rate per 1,000 person-years | 12.08 | 7.73 | 8.11 |  |
|  |  |  |  |  |
| Hazard ratio (95% CI)^b^ | 1.00 | 0.65 (0.46-0.91) | 0.65 (0.46-0.93) | 0.09 |
| Note: WHI OPACH participants enrolled in 2012-2013 and followed-up for cancer incidence through March 31, 2018. Women were censored at the end of follow-up, at last contact, or at the time of death from an event unrelated to cancer.  SD, standard deviation; BMI, body mass index; MVPA, moderate-to-vigorous physical activity; PA, physical activity.  ^a^ P-values from Chi-square tests for linear trend using Cox regression models with continuous PA variables.  ^b^ Model is adjusted for age (years), race/ethnicity (non-Hispanic white, non-Hispanic black, Hispanic), education (≤high school, some college, ≥college), and history of cancer diagnosis (no, yes), alcohol intake (non-drinker, <1 drink per week, 1-4 drinks per week, 5-7 drinks per week), hormone replacement therapy (no, yes), self-rated general health (excellent or very good, good, fair or poor), number of comorbid conditions (0, 1, 2, 3+), HEI-2010 (quartiles), and history of cancer diagnosis (no, yes). | | | | |

### **Supplemental Table 2**. Hazard ratios (HR) and 95% confidence intervals (CI) for physical activity-related cancer incidence and tertiles of wear time-standardized total, light, and moderate-to-vigorous physical activity, excluding women with censoring within the first six months of follow-up (*n*=6,323).

| **Total Physical Activity** | **Tertiles of Total PA** | | | ***P*_Trend_**^a^ |
| --- | --- | --- | --- | --- |
|  | **T1 (low)** | **T2** | **T3 (high)** |  |
| Total PA minutes per day, mean (SD) | 236.2 (43.4) | 331.7 (22.8) | 434.2 (52.0) |  |
|  |  |  |  |  |
| Cancer events, n (%) | 93 (4.4) | 75 (3.6) | 70 (3.3) |  |
| Person-years | 9,005.42 | 9,514.55 | 9,738.57 |  |
| Incidence rate per 1,000 person-years | 10.33 | 7.88 | 7.19 |  |
|  |  |  |  |  |
| Hazard ratio (95% CI)^b^ | 1.00 | 0.81 (0.59-1.10) | 0.75 (0.54-1.04) | 0.05 |
|  |  |  |  |  |
| **Light Physical Activity** | **Tertiles of Light PA** | | | ***P*_Trend_**^a^ |
|  | **T1 (low)** | **T2** | **T3 (high)** |  |
| Light PA minutes per day, mean (SD) | 205.6 (35.7) | 283.3 (18.1) | 364.3 (41.0) |  |
|  |  |  |  |  |
| Cancer events, n (%) | 89 (4.2) | 76 (3.6) | 73 (3.4) |  |
| Person-years | 9,084.03 | 9,518.68 | 9,655.83 |  |
| Incidence rate per 1,000 person-years | 9.80 | 8.0 | 7.56 |  |
|  |  |  |  |  |
| Hazard ratio (95% CI)^b^ | 1.00 | 0.86 (0.63-1.18) | 0.83 (0.60-1.14) | 0.15 |
|  |  |  |  |  |
| **Moderate-to-Vigorous Physical Activity** | **Tertiles of MVPA** | | | ***P*_Trend_**^a^ |
|  | **T1 (low)** | **T2** | **T3 (high)** |  |
| MVPA minutes per day, mean (SD) | 18.3 (8.7) | 43.3 (7.5) | 87.3 (27.3) |  |
|  |  |  |  |  |
| Cancer events, n (%) | 98 (4.7) | 68 (3.2) | 72 (3.4) |  |
| Person-years | 8,945.52 | 9,495.96 | 9,817.06 |  |
| Incidence rate per 1,000 person-years | 11.0 | 7.16 | 7.33 |  |
|  |  |  |  |  |
| Hazard ratio (95% CI)^b^ | 1.00 | 0.66 (0.48-0.91) | 0.67 (0.48-0.95) | 0.03 |
| Note: WHI OPACH participants enrolled in 2012-2013 and followed-up for cancer incidence through March 31, 2018. Women were censored at the end of follow-up, at last contact, or at the time of death from an event unrelated to cancer.  SD, standard deviation; BMI, body mass index; MVPA, moderate-to-vigorous physical activity; PA, physical activity.  ^a^ P-values from Chi-square tests for linear trend using Cox regression models with continuous PA variables.  ^b^ Model is adjusted for age (years), race/ethnicity (non-Hispanic white, non-Hispanic black, Hispanic), education (≤high school, some college, ≥college), smoking status/pack-years (never smoker; former smoker/≤7.5 or >7.5 pack-years; current smoker/≤22.5 or >22.5 pack-years, alcohol intake (non-drinker, <1 drink per week, 1-4 drinks per week, 5-7 drinks per week), hormone replacement therapy (no, yes), self-rated general health (excellent or very good, good, fair or poor), number of comorbid conditions (0, 1, 2, 3+), HEI-2010 (quartiles), and history of cancer diagnosis (no, yes). | | | | |

Supplemental Table 3. Hazard ratios (HRs) and 95% confidence intervals (CIs) for overall cancer incidence and tertiles of wear time-standardized total, light, and moderate-to-vigorous physical activity (*N*=6,382).

| **Total Physical Activity** | **Tertiles of Total PA** | | | ***P*_Trend_**^a^ |
| --- | --- | --- | --- | --- |
|  | **T1 (low)** | **T2** | **T3 (high)** |  |
| Cancer events, n (%) | 458 (21.6) | 396 (18.6) | 334 (15.7) |  |
| Person-years | 9,004.50 | 9,474.39 | 9,664.35 |  |
| Incidence rate per 1,000 person-years | 50.86 | 41.80 | 34.56 |  |
|  |  |  |  |  |
| **Model 1:** Hazard ratio (95% CI)^b^ | 1.00 | 0.79 (0.69-0.91) | 0.65 (0.56-0.75) | <0.01 |
| **Model 2:** Hazard ratio (95% CI)^c^ | 1.00 | 0.95 (0.83-1.09) | 0.89 (0.77-1.03) | 0.01 |
| **Model 3:** Hazard ratio (95% CI)^d^ | 1.00 | 0.95 (0.82-1.09) | 0.89 (0.76-1.04) | 0.02 |
|  |  |  |  |  |
| **Light Physical Activity** | **Tertiles of Light PA** | | | ***P*_Trend_**^a^ |
|  | **T1 (low)** | **T2** | **T3 (high)** |  |
| Cancer events, n (%) | 450 (21.2) | 382 (18.0) | 356 (16.7) |  |
| Person-years | 9,086.88 | 9,459.29 | 9,597.08 |  |
| Incidence rate per 1,000 person-years | 49.52 | 40.38 | 37.09 |  |
|  |  |  |  |  |
| **Model 1:** Hazard ratio (95% CI)^b^ | 1.00 | 0.78 (0.68-0.89) | 0.72 (0.63-0.83) | <0.01 |
| **Model 2:** Hazard ratio (95% CI)^c^ | 1.00 | 0.96 (0.83-1.10) | 0.91 (0.79-1.05) | 0.06 |
| **Model 3:** Hazard ratio (95% CI)^d^ | 1.00 | 0.95 (0.83-1.10) | 0.91 (0.79-1.06) | 0.07 |
|  |  |  |  |  |
| **Moderate-to-Vigorous Physical Activity** | **Tertiles of MVPA** | | | ***P*_Trend_**^a^ |
|  | **T1 (low)** | **T2** | **T3 (high)** |  |
| Cancer events, n (%) | 463 (21.8) | 398 (18.7) | 327 (15.3) |  |
| Person-years | 8,925.68 | 9,464.64 | 9,752.93 |  |
| Incidence rate per 1,000 person-years | 51.87 | 42.05 | 33.53 |  |
|  |  |  |  |  |
| **Model 1:** Hazard ratio (95% CI)^b^ | 1.00 | 0.79 (0.69-0.90) | 0.61 (0.52-0.71) | <0.01 |
| **Model 2:** Hazard ratio (95% CI)^c^ | 1.00 | 0.86 (0.75-0.98) | 0.81 (0.69-0.95) | 0.01 |
| **Model 3:** Hazard ratio (95% CI)^d^ | 1.00 | 0.86 (0.75-0.99) | 0.82 (0.70-0.96) | 0.01 |
| Note: WHI OPACH participants enrolled in 2012-2013 and followed-up for cancer incidence through March 31, 2018. Women were censored at the end of follow-up, at last contact, or at the time of death from an event unrelated to cancer.  BMI, body mass index; MVPA, moderate-to-vigorous physical activity; PA, physical activity; SD, standard deviation.  ^a^ P-values from Chi-square tests for linear trend using Cox regression models with continuous PA variables.  ^b^ Model 1 is adjusted for age (years).  ^c^ Model 2 is adjusted for age (years), race/ethnicity (non-Hispanic white, non-Hispanic black, Hispanic), education (≤high school, some college, ≥college), smoking status/pack-years (never smoker; former smoker/≤7.5 or >7.5 pack-years; current smoker/≤22.5 or >22.5 pack-years), alcohol intake (non-drinker, <1 drink per week, 1-4 drinks per week, 5-7 drinks per week), hormone replacement therapy (no, yes), self-rated general health (excellent or very good, good, fair or poor), number of comorbid conditions (0, 1, 2, 3+), HEI-2010 (quartiles), and history of cancer diagnosis (no, yes).  ^d^ Model 3 is adjusted for age (years), race/ethnicity (non-Hispanic white, non-Hispanic black, Hispanic), education (≤high school, some college, ≥college), smoking status/pack-years (never smoker; former smoker/≤7.5 or >7.5 pack-years; current smoker/≤22.5 or >22.5 pack-years), alcohol intake (non-drinker, <1 drink per week, 1-4 drinks per week, 5-7 drinks per week), hormone replacement therapy (no, yes), self-rated general health (excellent or very good, good, fair or poor), number of comorbid conditions (0, 1, 2, 3+), HEI-2010 (quartiles), history of cancer diagnosis (no, yes), and BMI (<18.5, 18.5-24.9, 25.0-29.9, 30-34.9, 35-39.9, ≥40 kg/m^2^). | | | | |

Supplemental Table 4. Hazard ratios (HR) and 95% confidence intervals (CI) for physical activity-related cancer incidence and standardized total, light, and moderate-to-vigorous physical activity in the cohort overall and after the exclusion of each cancer site at a time (*N*=6,382).

| **Cancer Site Excluded** | **Cancer Events**  ***n* (%)** | **Total PA^a^** |  | **Light PA^a^** |  | **MVPA^a^** |  |
| --- | --- | --- | --- | --- | --- | --- | --- |
|  |  | **HR (95% CI)^b^** |  | **HR (95% CI)^b^** |  | **HR (95% CI)^b^** |  |
| Breast | 173 | 0.87 (0.74-1.03) |  | 0.90 (0.77-1.06) |  | 0.85 (0.70-1.02) |  |
| Lung | 220 | 0.80 (0.69-0.93) |  | 0.84 (0.73-0.96) |  | 0.80 (0.68-0.94) |  |
| Colorectal | 239 | 0.88 (0.76-1.01) |  | 0.90 (0.79-1.03) |  | 0.86 (0.74-1.01) |  |
| Leukemia | 252 | 0.90 (0.78-1.02) |  | 0.92 (0.81-1.05) |  | 0.88 (0.76-1.02) |  |
| Bladder | 255 | 0.87 (0.76-0.99) |  | 0.89 (0.79-1.02) |  | 0.86 (0.75-1.00) |  |
| Multiple myeloma | 256 | 0.86 (0.75-0.98) |  | 0.89 (0.78-1.01) |  | 0.85 (0.73-0.99) |  |
| Endometrium | 262 | 0.86 (0.76-0.98) |  | 0.90 (0.79-1.02) |  | 0.84 (0.72-0.97) |  |
| Kidney | 265 | 0.89 (0.78-1.01) |  | 0.92 (0.81-1.04) |  | 0.86 (0.74-1.00) |  |
| Liver | 266 | 0.87 (0.77-1.00) |  | 0.91 (0.80-1.03) |  | 0.85 (0.73-0.98) |  |
| Stomach | 267 | 0.88 (0.77-1.00) |  | 0.91 (0.80-1.03) |  | 0.85 (0.74-0.99) |  |
| Head and neck | 267 | 0.87 (0.77-1.00) |  | 0.91 (0.80-1.03) |  | 0.85 (0.74-0.98) |  |
| Esophagus | 270 | 0.87 (0.77-0.99) |  | 0.90 (0.80-1.03) |  | 0.85 (0.73-0.98) |  |
| Note: WHI OPACH participants enrolled in 2012-2013 and followed-up for cancer incidence through March 31, 2018. Women were censored at the end of follow-up, at last contact, or at the time of death from an event unrelated to cancer.  ^a^ One-standard deviation unit increment of total PA = 90.8 minutes, light PA = 72.8 minutes, and MVPA = 33.3 minutes.  ^b^ Model is adjusted for age (years), race/ethnicity (non-Hispanic white, non-Hispanic black, Hispanic), education (≤high school, some college, ≥college), smoking status/pack-years (never smoker; former smoker/≤7.5 or >7.5 pack-years; current smoker/≤22.5 or >22.5 pack-years), alcohol intake (non-drinker, <1 drink per week, 1-4 drinks per week, 5-7 drinks per week), hormone replacement therapy (no, yes), self-rated general health (excellent or very good, good, fair or poor), number of comorbid conditions (0, 1, 2, 3+), HEI-2010 (quartiles), and history of cancer diagnosis (no, yes). | | | | | | | |
